# Feasibility Study of Multiorgan Dosiomics for Evaluating Radiation-Induced Xerostomia and Dysphagia in Head and Neck Cancer Radiotherapy

**DOI:** 10.3390/cancers18040619

**Published:** 2026-02-13

**Authors:** Takahiro Nakamoto, Koichi Yasuda, Takaaki Yoshimura, Takahiro Kanehira, Hiroshi Tamura, Sora Takagi, Sora Kobayashi, Tomohiko Miyazaki, Shuhei Takahashi, Yoshihiro Fujita, Takayuki Hashimoto, Hidefumi Aoyama

**Affiliations:** 1 Department of Biomedical Science and Engineering, Faculty of Health Sciences, Hokkaido University, N12-W5, Kita-ku, Sapporo 060-0812, Japan; 2 Department of Radiation Oncology, Hokkaido University Hospital, N14-W5, Kita-ku, Sapporo 060-8648, Japan; 3 Department of Health Sciences and Technology, Faculty of Health Sciences, Hokkaido University, N12-W5, Kita-ku, Sapporo 060-0812, Japan; 4 Department of Medical Physics, Hokkaido University Hospital, N14-W5, Kita-ku, Sapporo 060-8648, Japan; 5 Department of Radiological Technology, Hokkaido University Hospital, N14-W5, Kita-ku, Sapporo 060-8648, Japan; 6 Graduate School of Biomedical Science and Engineering, Hokkaido University, N15-W7, Kita-ku, Sapporo 060-8638, Japan; 7 Global Center for Biomedical Science and Engineering, Faculty of Medicine, Hokkaido University, N15-W7, Kita-ku, Sapporo 060-8638, Japan; 8 Department of Radiation Oncology, Faculty of Medicine, Hokkaido University, N15-W7, Kita-ku, Sapporo 060-8638, Japan

**Keywords:** dosiomics, radiotherapy, head and neck cancer, radiation-induced toxicity, xerostomia, dysphagia, multiple organs at risk

## Abstract

**Background/Objectives:** The severity of radiation-induced toxicity in head and neck cancer (HNC) radiotherapy should be predicted for the prognosis of the patient’s quality of life. Multiple organs at risk (OARs) are susceptible to toxicity in the head and neck. Hence, we aimed to investigate the feasibility of evaluating radiation-induced xerostomia and dysphagia based on multi-OAR dosiomics in HNC radiotherapy. **Methods:** We used radiotherapy treatment planning and toxicity data collected from 44 patients with HNC. High- and low-toxicity grades were classified using dosiomic models derived from multiple OARs. Dosiomic features were computed from the planned dose distribution per OAR. A prediction model was derived using selected dosiomic features and toxicity grades based on extreme gradient boosting for every OAR and all OARs. The model performance was evaluated in terms of the area under the curve (AUC) from leave-one-out cross-validation. Models based on dose volume histogram features and combining these with dosiomic features were derived to compare the prediction performance per OAR. Performance comparisons across OARs were also conducted. **Results:** The prediction models with the highest AUCs for xerostomia and dysphagia were the dosiomic model using all OARs, with an AUC of 0.843 (95% confidence interval—CI, 0.725–0.961), and that using the middle pharyngeal constrictor muscle, with an AUC of 0.878 (95% CI, 0.772–0.984). **Conclusions:** The evaluation results demonstrated the feasibility and potential of predicting radiation-induced toxicities based on multi-OAR dosiomics in HNC radiotherapy. Further investigations are required to determine the generalizability of our findings.

## Simple Summary

Radiation-induced toxicity is a serious concern in head and neck cancer (HNC) radiotherapy because multiple organs related to the quality of life (QOL) of patients are located in that region. Hence, toxicities must be assessed to manage the QOL during and after HNC radiotherapy. Dosiomics is a novel dosimetric analysis method for evaluating toxicity. In this study, we conducted a dosiomic analysis of HNC radiotherapy to comprehensively investigate the relation between the severity of toxicities and dosimetric features, including dose textures, in multiple organs at risk (OARs). We considered xerostomia and dysphagia as common toxicities in HNC radiotherapy. A dosiomic prediction model was built and validated for each OAR. The intra- and inter-OAR model performances were compared, demonstrating the feasibility of multi-OAR dosiomics for predicting radiation-induced toxicities in HNC radiotherapy. Our findings can provide a basis for future multiorgan dosiomic studies on toxicities in HNC radiotherapy.

## 1. Introduction

Head and neck cancer (HNC), especially squamous cell cancer, occurs at various sites in organs of the head and neck, mostly including the oral cavity, pharynx, and larynx [1,2]. Head and neck organs participate in eating, breathing, verbal communication, and other important functions that directly affect the quality of life (QOL) [3,4]. Ideally, in treatment policies, HNC should be controlled while sparing surrounding organs to ensure the high QOL of patients during and after treatment.

Radiotherapy, mostly in combination with chemotherapy (i.e., chemoradiotherapy), is a well-established treatment for HNC [5,6,7,8]. In particular, high-precision radiotherapy, such as intensity-modulated radiotherapy, can deliver doses that fit the tumors, being widely applied to treat HNC [5,7,8]. In addition, HNC control through radiotherapy preserves the surrounding normal organs and tissues [9]. However, it remains challenging to prevent functional decline in normal structures due to dose exposure [10]. Dermatitis, mucositis, dysgeusia, and mandibular osteoradionecrosis—especially xerostomia and dysphagia—are common radiation-induced toxicities in HNC radiotherapy and seriously affect the QOL of patients [10,11,12,13]. Thus, the occurrence and severity of these conditions should be predicted during radiation treatment planning to deliver dose distributions while considering the toxicities and facilitate the preparation of toxicity management protocols.

Analyzing statistical dosimetric parameters of treatment planning, including those of the dose volume histogram (DVH), is common for evaluating treatment outcomes in radiotherapy. In fact, the effectiveness of models to determine the DVH-based normal tissue complication probability regarding the risk of toxicity has been demonstrated in radiotherapy [14,15,16]. In HNC radiotherapy, dosimetric analyses have been conducted to assess radiation-induced xerostomia and dysphagia, determining relations between toxicities and dosimetric parameters of surrounding normal structures, often including clinical characteristics [17,18,19,20]. Nevertheless, the spatial texture characteristics of dose distributions within the objective structures cannot be considered for evaluation in such analysis [21] because the parameters are derived from first-order statistical measures related to the dose. Dose-based radiomics, known as dosiomics, can overcome the limitations of conventional dosimetric analysis by using dose spatial texture features.

Dosiomic analysis leverages quantitative radiomic features derived from planned dose distributions [22,23]. It has been widely applied to evaluate radiation-induced toxicity at various sites in radiotherapy [24,25,26,27]. In HNC treatment, dosiomic studies on building and evaluating xerostomia classification models have indicated potential for predicting xerostomia [28,29,30]. However, no study has been conducted on dysphagia prediction using dosiomics. Existing dosiomic studies on xerostomia have been focused on dose distributions within the parotid glands [28,29,30]. Although impairment of the parotid glands should be considered because they generate more than half of the saliva during activation [31], other salivary glands, such as the submandibular, sublingual, and oral cavity minor glands, must also be included in xerostomia dosiomic analysis. A model combining dosiomic features from multiple glands may improve the prediction accuracy compared with that from a single gland. Dosiomics for xerostomia should be applied to validate the predictive capabilities across salivary glands, individually and collectively. Swallowing is a complicated motor activity including multiple structures rather than a single organ [32]; therefore, this aspect should also be considered in the dosiomic analysis of dysphagia.

In this study, we aimed to investigate the feasibility of multiorgan dosiomics for evaluating radiation-induced xerostomia and dysphagia in HNC radiotherapy.

## 2. Materials and Methods

### 2.1. Study Design

**Figure 1:**
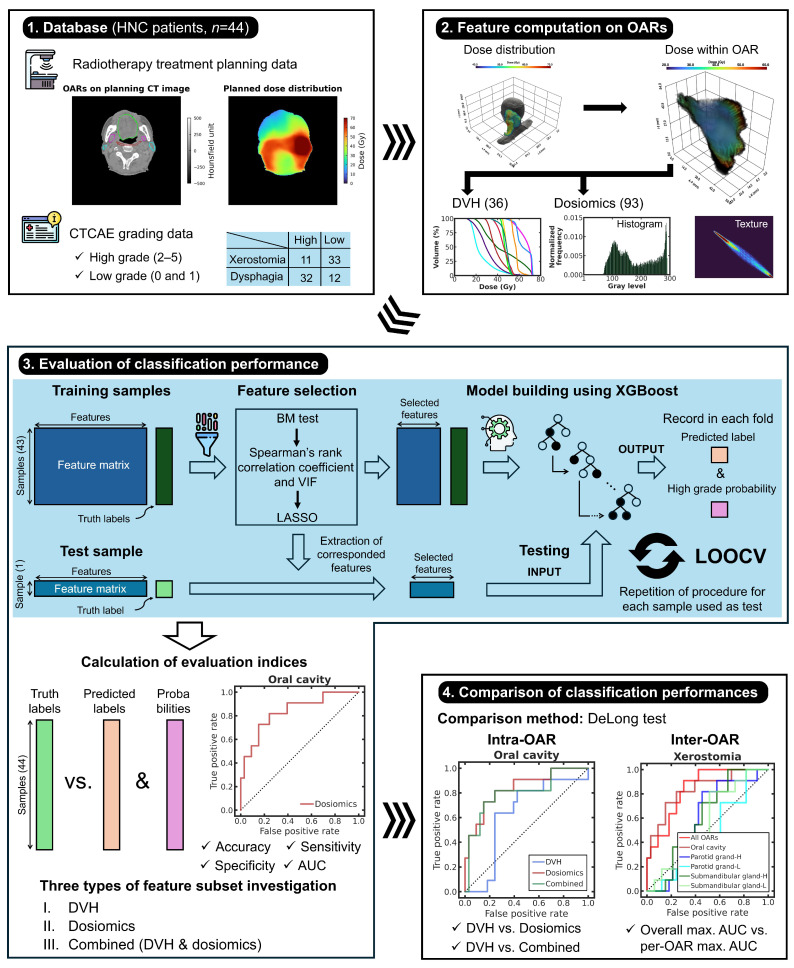
Schematic of study design comprising four steps: (1) collection of radiotherapy treatment planning and CTCAE grading data of 44 samples, (2) computation of dosimetric (i.e., DVH and dosiomic) features on OARs, (3) evaluation of XGBoost-based classification performance using LOOCV (blue shaded box), and (4) classification performances from intra- and inter-OAR comparisons. The number of OARs for xerostomia and dysphagia was five and six, respectively. DVH-, dosiomic-, and combined DVH/dosiomic-based classifications were executed for every OAR and for all OARs per toxicity. (Icons in steps 1 and 3 were sourced from https://www.freepik.com). To identify color references in this figure, the reader is referred to the web version of this article.

Figure 1 shows a schematic of the study design. We used 44 data records from patients with HNC who were treated with radiotherapy. Acute radiation-induced xerostomia and dysphagia were the targets for classification. The severities of xerostomia and dysphagia were evaluated by a radiation oncologist and binarized into high and low grades. Dosiomic and DVH features were computed from planned dose distributions on the various organs at risk (OARs) related to xerostomia and dysphagia. An extreme gradient boosting (XGBoost)-based supervised classification model was built using the selected features and high- and low-grade labels. DVH, dosiomics, and combined models were built per OAR. Leave-one-out cross-validation (LOOCV) was used to evaluate the prediction performances of the three classification models per OAR and to perform intra- and inter-OAR comparisons. The study was designed using an in silico approach. The computational environment and software libraries used in this study are described in Method S1 and Table S1.

### 2.2. Patient Characteristics

This study was conducted in accordance with the human ethics protocol approved by the Institutional Review Board of Hokkaido University Hospital (approval number 019-0397). We retrospectively enrolled 82 patients with HNC who were treated with radiotherapy between 2020 and 2022 at our hospital. We included patients who satisfied the following criteria: (i) HNC except for early glottic cancer, (ii) use of intensity-modulated photon radiotherapy, particularly volumetric modulated arc therapy with simultaneous integrated boost, (iii) prescribed dose of 70 Gy/35 Fr, (iv) completed therapy, and (v) no treatment replanning during therapy. After applying these criteria, 44 patients were included in this study. Table 1 lists the characteristics of the enrolled patients with HNC. All patients presented squamous cell carcinoma as the histological HNC subtype. One patient was a recurrent case, but radiotherapy was not used in their initial treatment. The degrees of acute xerostomia and dysphagia within 90 days after radiotherapy onset for the 44 patients were determined by an experienced HNC radiation oncologist (K.Y.) based on the common terminology criteria for adverse events (CTCAE) version 5.0 [33]. The CTCAE uses a five-point scale to assess adverse events (grades 1–5), with grade 0 indicating no observed adverse events. We categorized grades 2–5 as high (positive label) and grades 0 and 1 as low (negative label) for xerostomia and dysphagia.

### 2.3. Radiotherapy Treatment Planning and Delivery

Double-arc volumetric modulated arc therapy was applied to the 44 patients with HNC enrolled in this study. Planning computed tomography (CT) images were acquired using a single CT scanner (SOMATOM Confidence RT Pro, Siemens Healthineers, Erlangen, Germany) with a pixel size of 0.98 mm, matrix size of 512 × 512, slice thickness of 1 or 2 mm, and filtered back-projection reconstruction. Treatment plans were generated using three radiotherapy treatment planning systems: Pinnacle version 14.0 (Philips Healthcare, Amsterdam, The Netherlands) and RayStation versions 9A and 10A (RaySearch Laboratories AB, Stockholm, Sweden). Oncologists and medical physicists performed contouring of the target volumes and OARs and calculated the dose distributions, respectively, using the planning systems. The high-risk clinical target volume included the primary tumor, cervical lymph node metastases, and surrounding high-risk regions. The low-risk clinical target volume comprised the cervical prophylactic nodal region [34]. The high- and low-risk planning target volumes were generated by adding a margin to the clinical target volumes. Metal artifact regions due to dental silver fillings on the planning CT images were replaced with water-equivalent media before dose calculation. The dose distributions were calculated using inverse planning at a beam energy of 6 MeV using adaptive convolve in Pinnacle and collapse cone in RayStation (both superposition/convolution-type algorithms) and a grid size of 2 mm. The dose prescriptions were 70 Gy/35 Fr (2 Gy/Fr) to the high-risk planning target volume and 56 Gy/35 Fr (1.6 Gy/Fr) to the low-risk planning target volume at a dose covered by 95% of the planning target volumes with simultaneous integrated boost. In some plans, beam irradiation in an approximately anterior–posterior direction was skipped during arc motion owing to various factors related to dose optimization. The planned dose distributions were delivered using a linear accelerator (TrueBeam, Varian Medical System, Palo Alto, CA, USA). Data of planning CT images, OAR delineations, and dose distributions were exported in the DICOM-RT (Digital Imaging and Communications in Medicine for Radiotherapy) format from the planning systems for in silico analysis.

### 2.4. OARs of Xerostomia and Dysphagia for Analysis

Multiple OARs associated with xerostomia and dysphagia were investigated in this dosiomic study. As salivary gland impairment leads to xerostomia [12], we targeted dose distributions within the parotid and submandibular glands, as well as the oral cavities with minor salivary glands, for analysis to classify high and low grades. For dysphagia, we selected the superior, middle, and inferior pharyngeal constrictor muscles (PCMs), the cricopharyngeal muscle, and the supraglottic and glottic larynx, all of which are related to swallowing [35]. All targeted OARs in this study were delineated on the CT images by oncologists during treatment planning. Contouring was performed on the left and right sides of the parotid and submandibular glands. In each sample, the left and right glands were labeled as having high and low doses according to their mean doses. This labeling was applied after calculating the DVH features to consider the ipsilateral and contralateral glands with respect to the high-dose regions for feature analysis. We defined the sides of glands with high and low doses as gland-H and gland-L, respectively.

### 2.5. Dosiomic and DVH Features

Dosiomic and DVH features were calculated from the dose distribution in every target OAR. Before feature calculations, processing involved aligning the dose distribution on the CT image, isotropically resampling the dose distribution and OAR, and discretizing the dose distribution on the OAR. Preprocessing was performed as detailed in Method S2. We obtained dosiomic features based on the differential histogram (20 features), gray-level (GL) cooccurrence matrix texture (22), GL run length matrix texture (16), GL size zone matrix texture (16), neighboring GL dependence matrix texture (14), and neighborhood gray-tone difference matrix texture (5). The parameters of maximum, minimum, and mean doses (in grays), doses (in grays) covered by *x*% of volume (D*x*, 19 parameters), and volumes (in percentages) receiving more than *x* Gy of dose (V*x*, 14 parameters) were calculated as DVH features. The *x* values of D*x* and V*x* were determined from 5% to 95% and from 5 to 70 Gy in increments of 5% and 5 Gy, respectively, and the features were calculated by DVH curves with 0.01 Gy bins. The 93 dosiomic and 36 DVH features are listed in Table S2, and the computation parameters of the texture matrices are listed in Table S3.

### 2.6. Modeling and Performance Evaluation of Classification Using LOOCV

#### 2.6.1. LOOCV Design

We employed LOOCV to evaluate the predictive ability of dosimetric features for toxicity grades, given the limited sample size. First, the data from the 44 patients were split into 43 training samples and 1 test sample. Second, dosimetric features were selected using the training samples. Third, an XGBoost-based classification model was built using the selected dosimetric features and toxicity grade labels from the training samples. Finally, the prediction label and predictive probability of obtaining a high grade in the test sample were recorded by inputting the selected dosimetric features of the test sample to the trained model. This procedure was repeated until each of the 44 samples was used as the test sample. The accuracy, sensitivity, specificity, and area under the empirical receiver operating characteristic (ROC) curve (AUC) were calculated from the true toxicity label, LOOCV-derived prediction labels, and probabilities across samples to evaluate the classification performance. We divided the dosimetric feature set into (i) dosiomic, (ii) DVH, and (iii) combined DVH/dosiomic features. LOOCV was applied to the three types of features per OAR and for all OARs associated with xerostomia and dysphagia to compare the intra- and inter-OAR classification performance.

#### 2.6.2. Feature Selection

The feature selection strategy comprised four consecutive steps: (i) statistical filtering by Brunner–Munzel test, (ii) redundancy filtering by Spearman’s rank correlation coefficients, (iii) multicollinearity filtering by variance inflation factor (VIF), and (iv) regularization-based selection by least absolute shrinkage and selection operator (LASSO). During feature selection, if the number of selected features was zero at any step, that step was skipped. For statistical and redundancy filtering, we used the procedures and cutoffs described in [36]. To address multicollinearity among features after filtering, selection based on VIF was used. VIF is a multicollinearity score for a feature that is derived from linear regression between the feature as a response variable and the other features as explanatory variables [37]. The higher the VIF for a feature, the higher its multicollinearity. We repeated recursive elimination of features with the maximum VIF in the corresponding feature subset until the maximum VIF in that subset was less than or equal to 10 [38]. Finally, we selected features with nonzero weight coefficients in the LASSO model, which was built using the VIF-filtered features and toxicity grade labels. LASSO is an L1-norm regularized regression that builds a sparse model, where most model coefficients would be zero [39]. We applied *z*-score normalization to the features before building the LASSO model. The regularization parameter in LASSO was tuned by grid search based on mean-squared-error minimization with stratified five-time fivefold cross-validation. The search range of the regularization parameter was 0.01–1.00 in increments of 0.01. We fixed a random seed of 42 for shuffling samples in grid search cross-validation to ensure reproducibility.

#### 2.6.3. Model Building and Validation

An XGBoost-based classification model was built using training samples with selected features and toxicity grade labels. We applied *z*-score feature normalization and the synthetic minority oversampling technique to address imbalanced data before building the model. XGBoost is a nonlinear machine-learning algorithm that performs ensemble learning based on gradient boosting decision trees [40], with demonstrated reliability and effectiveness in various trials and competitions [41]. During model building, we optimized the following tree structure regularization hyperparameters: maximum depth of tree (max_depth), maximum number of leaf nodes (max_leaves), and minimum loss reduction to split a node (gamma) using grid search with AUC maximization and stratified five-time fivefold cross-validation. The search points in max_depth, max_leaves, and gamma were (2, 3), (2, 3), and (0.1, 1, 10), respectively. The remaining XGBoost hyperparameters were fixed to the default values of the computational library. The random seeds of grid search cross-validation, synthetic minority oversampling technique, and XGBoost were fixed as for LASSO in feature selection.

We fed the selected features of the test sample to the trained model for validation. Before inputting the test sample into the models, the selected features of the test sample were normalized using the *z*-score parameters determined from the training samples. The model outputs were the probabilities of obtaining positive and negative labels (complementary events), which corresponded to high and low grades, respectively. We set a cutoff in the positive probability to perform binary classification. The cutoff was determined based on the maximum Youden index of the model ROC curve for the training samples. If the probability reached or surpassed the cutoff, the prediction was high grade; otherwise, it was low grade. The prediction label and high-grade probability in the test sample were recorded across LOOCV rounds. Then, the prediction performance evaluation indices of accuracy, sensitivity, and specificity were calculated from the true labels and pooled LOOCV prediction labels in all samples. Moreover, the AUC was calculated by ROC curve analysis using true labels and pooled LOOCV high-grade probability.

### 2.7. Comparison of Classification Performance

We evaluated the classification performances using the statistical significance of AUCs of the intra- and inter-OAR comparisons by applying a two-sided DeLong test per toxicity. In the intra-OAR comparison, the DeLong test was applied to compare the AUCs of the dosiomics and combination against the DVH for each OAR and all OARs. In the inter-OAR comparison, the DeLong test was applied to compare the highest AUCs from the three feature subsets for each OAR and all OARs against the overall highest AUC. We set a significance threshold of 5% (*p* < 0.05) for statistical analysis. Bonferroni correction for the significance level was employed to control the false discovery rate (i.e., type I error) because multiple comparisons were conducted in the intra- and inter-OAR analyses. The corrected significance threshold for *p*-values in the intra-OAR comparison was 0.025 (=0.05/2), and that for inter-OAR comparisons for xerostomia and dysphagia was 0.01 (=0.05/5) and 0.833 × 10^−2^ (≃0.05/6), respectively.

## 3. Results

**Figure 2:**
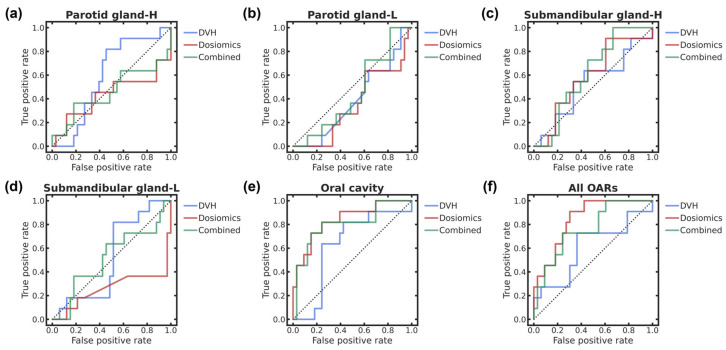
ROC curves of xerostomia classification for three dosimetric feature subsets in (**a**) parotid gland-H, (**b**) parotid gland-L, (**c**) submandibular gland-H, (**d**) submandibular gland-L, (**e**) oral cavity, and (**f**) all OARs. The dotted lines represent the baseline ROC curves with an AUC of 0.5. The blue, red, and green solid lines represent the ROC curves of classification with DVH, dosiomic, and combined DVH/dosiomic features, respectively. To identify color references in this figure, the reader is referred to the web version of this article.

**Figure 3:**
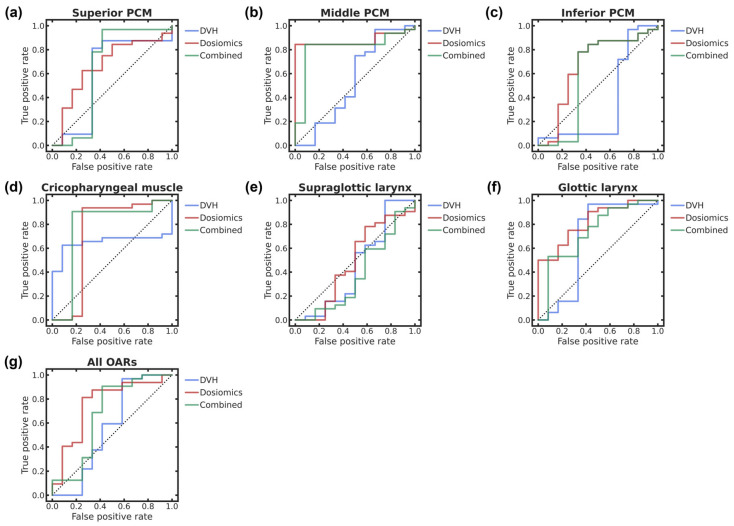
ROC curves of dysphagia classification performances with three dosimetric feature subsets in (**a**) superior, (**b**) middle, (**c**) and inferior PCMs, (**d**) cricopharyngeal muscle, (**e**) supraglottic larynx, (**f**) glottic larynx, and (**g**) all OARs. The dotted lines represent the baseline ROC curves with an AUC of 0.5. The blue, red, and green solid lines represent the ROC curves of classifications with DVH, dosiomic, and combined DVH/dosiomic features, respectively. To identify color references in this figure, the reader is referred to the web version of this article.

Table 2 and Table 3 list the evaluation indices related to the classification of xerostomia and dysphagia, respectively, with three dosimetric feature subsets per OAR and *p*-values of the intra- and inter-OAR performance comparisons. In addition, Figure 2 and Figure 3 show the ROC curves for xerostomia and dysphagia, respectively, with the feature subsets for each OAR and all OARs. The ROC curve analysis shows that the best performances for xerostomia and dysphagia are dosiomic-based classification of all OARs with the highest AUC of 0.843 (95% confidence interval—CI, 0.725–0.961) and dosiomic-based classification of middle PCM with the highest AUC of 0.878 (95% CI, 0.772–0.984), respectively. In the intra-OAR comparisons, statistically significant differences in the AUCs occur between the DVH and dosiomic features in all OARs for xerostomia and the middle and inferior PCMs for dysphagia (*p* < 0.025). In the inter-OAR comparisons, statistically significant differences are observed in the highest AUCs of feature subsets in the parotid gland-L as well as submandibular glands-H and -L across OARs, with the overall highest AUC being the reference for xerostomia (*p* < 0.010) and the supraglottic larynx from the middle PCM with the overall highest AUC being the reference for dysphagia (*p* < 0.833 × 10^−2^).

## 4. Discussion

We conducted a dosiomic analysis to predict acute radiation-induced complications in HNC radiotherapy, considering multiple OARs, including various glands, muscles, and organs related to xerostomia and dysphagia. Moreover, the predictive performance of three dosimetric feature subsets was investigated per OAR for subsequent intra- and inter-OAR comparisons. Prediction models derived considering all OARs related to xerostomia and dysphagia were also investigated. This was the first attempt to comprehensively investigate the relation between various dosimetric features from multiple OARs and complications in HNC radiotherapy.

Estimating middle and late radiation-induced toxicities is crucial for assessing the QOL of patients in the long term after HNC radiotherapy. Similarly, acute toxicity evaluation is important for estimating QOL during and immediately after treatment. In addition, Dean et al. [42] noted that the severity of acute radiation-induced toxicity responses is associated with late toxicities [43,44]. Thus, predicting acute toxicities may facilitate radiation treatment replanning and allow for the consideration of management strategies for these toxicities in advance, thereby preventing the development of late toxicities. Therefore, acute radiation-induced head and neck organ toxicities were targeted for prediction in this study.

Several studies on the prediction of acute xerostomia during HNC radiotherapy or within 3-month follow-ups have been conducted by using texture, radiomic, and dosiomic analyses in the major salivary glands [30,45,46,47]. Those studies assumed that dysfunctions of the parotid or submandibular glands are the main drivers of acute xerostomia. Similarly, we investigated the feasibility of predicting acute xerostomia based on the dosimetric features of glands. Although there were differences between those studies and our work regarding the analysis strategy and data size, the AUC ranges of 0.262–0.612 for parotid gland-H and -L and submandibular gland-H and -L in our results indicate that dosimetric features in the major salivary glands are not meaningful predictors of acute xerostomia, contradicting the common assumption.

The best prediction performance in acute xerostomia was achieved by the dosiomic model of all OARs, with an AUC of 0.843 (95% CI, 0.725–0.961). This comprehensive model significantly outperformed some models that considered a single OAR. Although using various combinations of multi-OAR dosiomic features to build the model improved the prediction performance, the mean and standard deviation percentages of oral cavity features in the feature subset for building the model across LOOCV folds were 53.2% ± 9.6%. This indicated that features derived from the oral cavity were major predictors for the model. The dosiomic model of the oral cavity had the second-best prediction performance, with an AUC of more than 0.80 and no significant difference from the dosiomic model of all OARs. Thus, the dose on the oral cavity might be essential for assessing the severity of acute radiation-induced xerostomia.

The oral cavities in dosimetric analysis have been mostly neglected because they have minor salivary glands. In fact, Fried et al. [48] found scarce studies on xerostomia considering dosimetric analysis in the oral cavity, and the available studies showed inconsistent findings (e.g., [49,50]). The reliability of our findings may be limited because this is the first dosiomic analysis of the oral cavity. Further dosiomic investigations with more samples and various validation methods remain to be conducted to confirm our findings for the oral cavity.

During analysis of acute dysphagia, the best prediction performance was achieved by the dosiomic model of the middle PCM, with an AUC of 0.878 (95% CI, 0.772–0.984). Although the AUC was approximately 0.90 in the model, no statistically significant differences were observed in the inter-OAR comparison, except for the model of the supraglottic larynx. Therefore, we could not identify the most relevant OAR for predicting acute dysphagia in the conducted dosimetric analysis. In addition, the effectiveness of using the features of all OARs was not confirmed. Hence, we can only suggest that dosimetric models of some OARs had lower AUC confidence limits exceeding 0.50, indicating potential for classifying acute dysphagia into high or low grade.

Several studies have been conducted on acute dysphagia evaluation during HNC radiotherapy or at least within 3-month follow-ups based on conventional dosimetric analysis [42,51,52]. Dean et al. built a machine-learning-based model for evaluating acute dysphagia using pharyngeal mucosa DVH parameters and investigated the model performance [42]. A dosimetric analysis of acute and late dysphagia conducted by Mazzola et al. indicated that the dose to the middle PCM might be related to acute dysphagia [51]. Alterio et al. reported that dose damage to the cricopharyngeal muscle and cervical esophagus might be associated with acute dysphagia [52]. Although the middle PCM demonstrated the best performance in our analysis, aligning with the findings by Mazzola et al. [51], variability in the results of acute dysphagia analysis may persist owing to the scarce studies available. Further research is necessary to obtain robust findings and unveil physiological interpretations.

Dosiomic- and DVH-based models were compared to evaluate the dosiomic approach in HNC radiotherapy. For xerostomia, the AUCs for dosiomics were lower than those for DVH for several OARs. No statistically significant differences were observed between the dosiomic- and DVH-based models per OAR. Therefore, we could not confirm the effectiveness of the dosiomic approach for xerostomia. However, for dysphagia, all dosiomic models outperformed the DVH models in terms of AUCs. The middle and inferior PCMs showed statistically significant differences between the dosiomic- and DVH-based models. Dosiomics may serve as an alternative dosimetric method for accurately assessing the severity of dysphagia. The comparison between DVH and combined DVH/dosiomic features revealed a similar pattern to that observed in the comparison between separate DVH and dosiomic features.

Dosiomic analysis is sensitive to variation in features owing to differences in planning parameters such as dose calculation grid sizes and algorithms. In this study, although the dose calculation grid size and algorithm were standardized across the database, we used data from three radiotherapy treatment planning systems. Characteristics in these different systems are propagated to dosiomic features and could affect the analysis. A harmonization technique [53] may be valid to mitigate the influence of different system characteristics (batch effect) in dosiomic features.

Dosiomic features are dosimetric parameters that include spatial dose texture information. Modern radiotherapy techniques, such as intensity-modulated radiotherapy, can deliver homogeneous doses to the targets [54,55]. Dosiomic features derived from targets likely exhibit similarity, even across patients, owing to the homogeneous dose. Therefore, dosiomics is suitable for addressing radiation-induced toxicities of OARs, as illustrated in this study, rather than target outcomes such as tumor control and prognosis. To date, dosiomic features and models have been mainly applied as evaluation tools or predictors of radiation-induced toxicity [24,25,26,27,28,29,30]. In this study, the feasibility of multiorgan dosiomics for predicting acute radiation-induced xerostomia and dysphagia in HNC radiotherapy to manage the QOL of patients has been suggested. In clinical practice, our framework could provide feedback on toxicity predictions to radiation oncologists and medical physicists, aiding them in treatment planning while considering toxicity. It also allows for the organization of toxicity management during and after treatment in HNC radiotherapy. As a next step, dosiomic features and models may be used to establish constraints on dose optimization to perform OAR sparing, considering spatial dose textures.

This study had some limitations. First, it was designated as a feasibility study due to the limited sample size. Although LOOCV was applied to handle analysis on a small dataset, the results might not ensure statistical stability. Our findings should be confirmed considering larger cohorts. In addition, dose uncertainties were propagated into the dosimetric features for analysis. In the dosimetric analysis of HNC, unavoidable dose uncertainties occurred owing to metal artifacts caused by dental silver fillings. Although the artifact regions were overridden with water-equivalent media during dose calculation, removing uncertainty remained challenging. A metal artifact reduction algorithm [56] may be used to address dose uncertainties in the dosimetric analysis of HNC.

## 5. Conclusions

We investigated multi-OAR-derived dosiomic models to predict the severity of acute radiation-induced xerostomia and dysphagia in HNC radiotherapy. The oral-cavity-derived dosiomic model seems effective for evaluating xerostomia. In dysphagia, although we could not specify a definitive OAR dosiomic model for accurate prediction, dosiomic models seem promising. In intra-OAR comparisons, the dosiomic-based approach outperformed the DVH-based approach in predicting dysphagia. Nevertheless, we could not confirm the effectiveness of combining all OARs in predicting each toxicity in the inter-OAR comparisons. Future work should be aimed at demonstrating the reliability of the findings from this study using large cohorts.

## Figures and Tables

**Table 1:** Characteristics of patients with HNC enrolled in this study.

Characteristic	Value
Number of cases, *n*	44
Median age, years (interquartile range)	66 (57–71)
Number of men, *n* (%)	34 (77.3)
Tumor site	
Nasopharynx, *n* (%)	11 (25.0)
Oropharynx, *n* (%)	17 (38.6)
Hypopharynx, *n* (%)	12 (27.3)
Larynx, *n* (%)	4 (9.1)
Clinical stage based on Union for International Cancer Control 8th edition	
I, *n* (%)	8 (18.2)
II, *n* (%)	13 (29.5)
III, *n* (%)	11 (25)
IV, *n* (%)	12 (27.3)
Treatment procedure	
Concurrent chemoradiotherapy (CCRT), *n* (%)	29 (65.9)
CCRT with induction chemotherapy, *n* (%)	2 (4.5)
CCRT with adjuvant chemotherapy, *n* (%)	3 (6.8)
Radiotherapy alone, *n* (%)	10 (22.7)
Xerostomia CTCAE grade	
Grade 0, *n* (%)	1 (2.3)
Grade 1, *n* (%)	32 (72.7)
Grade 2, *n* (%)	11 (25.0)
Xerostomia binarized grade in this study	
High grade (grades 2–5), *n* (%)	11 (25.0)
Low grade (grades 0 and 1), *n* (%)	33 (75.0)
Dysphagia CTCAE grade	
Grade 0, *n* (%)	6 (13.6)
Grade 1, *n* (%)	6 (13.6)
Grade 2, *n* (%)	6 (13.6)
Grade 3, *n* (%)	26 (59.1)
Dysphagia binarized grade in this study	
High grade (grades 2–5), *n* (%)	32 (72.7)
Low grade (grades 0 and 1), *n* (%)	12 (27.3)

**Table 2:** Evaluation indices of xerostomia classification for three dosimetric feature subsets per OAR and *p*-values of the intra- and inter-OAR performance comparisons.

OAR	Feature Subset	Accuracy	Sensitivity	Specificity	AUC (95% CI)	*p*-Value of Intra-OAR Comparison	*p*-Value of Inter-OAR Comparison
Parotid gland-H	DVH	0.659 (29/44)	0.545 (6/11)	0.697 (23/33)	**0.595 (0.418–0.772)**	Ref.	0.139 × 10^−1^
	Dosiomics	0.636 (28/44)	0.364 (4/11)	0.727 (24/33)	0.433 (0.191–0.674)	0.188	–
	Combined	0.636 (28/44)	0.364 (4/11)	0.727 (24/33)	0.460 (0.224–0.697)	0.249	–
Parotid gland-L	DVH	0.614 (27/44)	0.182 (2/11)	0.758 (25/33)	0.388 (0.212–0.565)	Ref.	–
	Dosiomics	0.614 (27/44)	0.273 (3/11)	0.727 (24/33)	0.350 (0.168–0.532)	0.735	–
	Combined	0.591 (26/44)	0.364 (4/11)	0.667 (22/33)	**0.449 (0.265–0.633)**	0.612	0.845 × 10^−3 †^
Submandibular gland-H	DVH	0.568 (25/44)	0.545 (6/11)	0.576 (19/33)	0.529 (0.320–0.738)	Ref.	–
	Dosiomics	0.636 (28/44)	0.364 (4/11)	0.727 (24/33)	0.584 (0.390–0.778)	0.601	–
	Combined	0.636 (28/44)	0.364 (4/11)	0.727 (24/33)	**0.612 (0.441–0.782)**	0.503	0.923 × 10^−2 †^
Submandibular gland-L	DVH	0.591 (26/44)	0.182 (2/11)	0.727 (24/33)	**0.521 (0.335–0.706)**	Ref.	0.741 × 10^−2 †^
	Dosiomics	0.568 (25/44)	0.091 (1/11)	0.727 (24/33)	0.262 (0.048–0.476)	0.769 × 10^−1^	–
	Combined	0.614 (27/44)	0.364 (4/11)	0.697 (23/33)	0.518 (0.308–0.728)	0.980	–
Oral cavity	DVH	0.659 (29/44)	0.727 (8/11)	0.636 (21/33)	0.628 (0.438–0.818)	Ref.	–
	Dosiomics	0.818 (36/44)	0.727 (8/11)	0.848 (28/33)	**0.837 (0.695–0.980)**	0.493 × 10^−1^	0.918
	Combined	0.818 (36/44)	0.727 (8/11)	0.848 (28/33)	0.802 (0.634–0.969)	0.114	–
All OARs	DVH	0.636 (28/44)	0.636 (7/11)	0.636 (21/33)	0.606 (0.390–0.823)	Ref.	–
	Dosiomics	0.727 (32/44)	0.455 (5/11)	0.818 (27/33)	**0.843 (0.725–0.961)**	0.143 × 10^−1^ *	Ref.
	Combined	0.727 (32/44)	0.545 (6/11)	0.788 (26/33)	0.763 (0.603–0.923)	0.512 × 10^−1^	–

* *p* < 0.025, ^†^ *p* < 0.01. “H” and “L” denote high and low doses, respectively. The value in bold is the highest AUC in the feature subsets per OAR. The AUC with DVH in each OAR is the reference (Ref.) for the DeLong test with a corrected significance cutoff of 0.025 (asterisk) in the intra-OAR comparisons. The AUC with dosiomics across OARs is the reference (Ref.) for the test with a corrected significance cutoff of 0.01 (dagger) in the inter-OAR comparisons. The en dashes in the inter-OAR comparisons indicate non-computable values.

**Table 3:** Evaluation indices of dysphagia classification for three dosimetric feature subsets per OAR and *p*-values of intra- and inter-OAR performance comparisons.

OAR	Feature Subset	Accuracy	Sensitivity	Specificity	AUC (95% CI)	*p*-Value of Intra-OAR Comparison	*p*-Value of Inter-OAR Comparison
Superior PCM	DVH	0.727 (32/44)	0.750 (24/32)	0.667 (8/12)	0.602 (0.363–0.840)	Ref.	–
	Dosiomics	0.614 (27/44)	0.625 (20/32)	0.583 (7/12)	**0.669 (0.480–0.858)**	0.374	0.532 × 10^−1^
	Combined	0.705 (31/44)	0.719 (23/32)	0.667 (8/12)	0.641 (0.380–0.901)	0.336	–
Middle PCM	DVH	0.636 (28/44)	0.688 (22/32)	0.500 (6/12)	0.544 (0.308–0.780)	Ref.	–
	Dosiomics	0.864 (38/44)	0.844 (27/32)	0.917 (11/12)	**0.878 (0.772–0.984)**	0.111 × 10^−1^ *	Ref.
	Combined	0.818 (36/44)	0.844 (27/32)	0.750 (9/12)	0.815 (0.663–0.967)	0.255 × 10^−1^	–
Inferior PCM	DVH	0.614 (27/44)	0.719 (23/32)	0.333 (4/12)	0.365 (0.119–0.611)	Ref.	–
	Dosiomics	0.773 (34/44)	0.813 (26/32)	0.667 (8/12)	**0.667 (0.453–0.880)**	0.212 × 10^−1^ *	0.745 × 10^−1^
	Combined	0.773 (34/44)	0.813 (26/32)	0.667 (8/12)	0.591 (0.340–0.842)	0.912 × 10^−1^	–
Cricopharyngeal muscle	DVH	0.636 (28/44)	0.656 (21/32)	0.583 (7/12)	0.651 (0.489–0.813)	Ref.	–
	Dosiomics	0.886 (39/44)	0.938 (30/32)	0.750 (9/12)	0.721 (0.474–0.968)	0.596	–
	Combined	0.886 (39/44)	0.906 (29/32)	0.833 (10/12)	**0.771 (0.555–0.987)**	0.343	0.404
Supraglottic larynx	DVH	0.568 (25/44)	0.625 (20/32)	0.417 (5/12)	0.453 (0.217–0.689)	Ref.	–
	Dosiomics	0.614 (27/44)	0.688 (22/32)	0.417 (5/12)	**0.487 (0.261–0.713)**	0.750	0.282 × 10^−2 †^
	Combined	0.523 (23/44)	0.563 (18/32)	0.417 (5/12)	0.383 (0.170–0.596)	0.492	–
Glottic larynx	DVH	0.682 (30/44)	0.688 (22/32)	0.667 (8/12)	0.667 (0.420–0.913)	Ref.	–
	Dosiomics	0.705 (31/44)	0.750 (24/32)	0.583 (7/12)	**0.820 (0.687–0.953)**	0.834 × 10^−1^	0.522
	Combined	0.682 (30/44)	0.719 (23/32)	0.583 (7/12)	0.732 (0.547–0.917)	0.452	–
All OARs	DVH	0.659 (29/44)	0.750 (24/32)	0.417 (5/12)	0.560 (0.313–0.807)	Ref.	–
	Dosiomics	0.773 (34/44)	0.844 (27/32)	0.583 (7/12)	**0.760 (0.581–0.940)**	0.791 × 10^−1^	0.260
	Combined	0.818 (36/44)	0.906 (29/32)	0.583 (7/12)	0.672 (0.448–0.896)	0.241	–

* *p* < 0.025, ^†^ *p* < 0.833 × 10^−2^. The value in bold is the highest AUC in the feature subsets per OAR. The AUC with DVH in each OAR is the reference (Ref.) for the DeLong test with a corrected significant cutoff of 0.025 (asterisk) in the intra-OAR comparisons. The AUC with dosiomics in the middle PCM is the reference (Ref.) for the test with a corrected significant cutoff of 0.833 × 10^−2^ (dagger) in the inter-OAR comparisons. The en dashes in the inter-OAR comparisons indicate non-computable values.

## Data Availability

Due to the nature of this research, participants of this study did not agree for their data to be shared publicly. Thus, patients’ data are not available.

## Supplementary Materials

The following supporting information can be downloaded at https://www.mdpi.com/article/10.3390/cancers18040619/s1. Method S1: Computational environment; Method S2: Preprocessing for feature calculation; Table S1: Computational libraries used in this study; Table S2: Dosimetric features used in this study; Table S3: Computation parameters of texture matrices used in this study.

## Abbreviations

The following abbreviations are used in this manuscript:

AUC	Area under the curve
CI	Confidence interval
CT	Computed tomography
CTCAE	Common terminology criteria for adverse events
DVH	Dose volume histogram
D*x*	Dose (in gray) covered by *x*% of volume
GL	Gray level
HNC	Head and neck cancer
LASSO	Least absolute shrinkage and selection operator
LOOCV	Leave-one-out cross-validation
OAR	Organ at risk
PCM	Pharyngeal constrictor muscle
QOL	Quality of life
ROC	Receiver operating characteristic
VIF	Variance inflation factor
V*x*	Volume (in percentage) receiving more than *x* Gy of dose
XGBoost	Extreme gradient boosting
