# Supplementary material for: Feasibility Study of Multiorgan Dosiomics for Evaluating Radiation-Induced Xerostomia and Dysphagia in Head and Neck Cancer Radiotherapy
Source: Cancers (Basel). 2026 Feb 13;18(4):619. doi: 10.3390/cancers18040619 (PMC12938932; doi:10.3390/cancers18040619)
Supplement: Supplementary file 1 [file cancers-18-00619-s001.zip › cancers-4113356-supplementary.pdf]

cancers MDPI

Supplementary Methods

Methods S1: Computational environment

We used a general-purpose workstation equipped with a multicore central processing unit (Intel Core i9-14900KF; Intel, Santa Clara, CA, USA) and 128 GB of random-access memory for this study. Table S1 lists the computational libraries used in this study. MATLAB version R2022b (MathWorks, Natick, MA, USA) was used for preprocessing and dosimetric feature extraction. We executed the MATLAB programs on a Windows 11 Pro operating system (Microsoft, Redmond, WA, USA). Python version 3.10 and R version 4.4 were used to perform dosimetric feature analysis. The R scripts were handled using Python with PypeR version 1.1. The Python and R scripts were executed on Ubuntu version 22.04 Long Term Support hosted through the Windows Subsystem for Linux version 2 (Microsoft, Redmond, WA, USA).

Methods S2: Preprocessing for feature calculation

Preprocessing comprised three steps: (i) alignment of dose distribution on the corresponding computed tomography (CT) image, (ii) isotropic resampling of the dose distribution and organ at risk (OAR), and (iii) discretization of dose distribution on the OAR.

Planned dose distributions in the radiotherapy treatment planning systems were exported as three-dimensional images with 2.0 × 2.0 × 2.0 mm^3 voxels (dose calculation grid size) in the DICOM-RT (Digital Imaging and Communications in Medicine for Radiotherapy) format. The dose distribution image data were expressed in the planning CT image coordinates because the contouring data of OARs were defined on the CT images. The transformation was executed by resampling the dose distributions with the same voxel size as the CT images using Lanczos3 interpolation and then representing the resampled dose distribution on the CT image coordinates based on the reference positions of the CT and dose distribution images.

To compute the dosimetric features with an isotropic voxel size, resampling of the dose distribution and OAR mask images was necessary. Lanczos3 interpolation was utilized for resampling the dose distribution images, while shape-based interpolation [1] with Lanczos3 was employed for resampling the OAR mask images. A resampling voxel of 1.0 × 1.0 × 1.0 mm^3 was used.

The dose distributions on the OARs were extracted by cropping the dose distribution images with OAR mask images. In dosiomics, dose distributions should be discretized to calculate differential histogram- and texture-based features. Thus, we employed a fixed bin size method [2] to discretize the dose distribution images because the image intensity represented the absorbed dose with its underlying physical meaning. In this study, the bin size and minimum (baseline) value were set to 0.25 and 0 Gy, respectively.

Supplementary Tables

Table S1. Computational libraries used in this study.

MATLAB			
**Library**	**Version**	**Purpose**	**Website**
Radiomics-develop [3,4]	–	Pre-processing, Feature calculation	https://github.com/mvallieres/radiomics-develop
**Python**			
**Library**	**Version**	**Purpose**	**Website**
Numpy	1.24	Numerical calculation	https://numpy.org
SciPy	1.10	Brunner–Munzel test	https://scipy.org
Pandas	1.5	Spearman's rank correlation coefficient calculation	https://pandas.pydata.org

scikit-learn	1.2	Leave-one-out cross-validation, variance inflation factor calculation, least absolute shrinkage and selection operator	https://scikit-learn.org
imbalanced-learn	0.10	Synthetic minority over-sampling technique	https://imbalanced-learn.org
XGBoost	1.7	Extreme gradient boosting	https://xgboost.readthedocs.io/en/latest/index.html
Seaborn	0.12	Graph visualization	https://seaborn.pydata.org
Matplotlib	3.6	Graph visualization	https://matplotlib.org
PypeR	1.1	R script execution via Python	https://pypi.org/project/PypeR
**R**			
**Library**	**Version**	**Purpose**	**Website**
pROC	1.18	Receiver operating characteristic analysis, DeLong test	https://cran.r-project.org/web/packages/pROC

Table S2. Dosimetric features used in this study including 20 differential histogram-based, 73 texture-based, and 36 dose volume histogram (DVH) features. The differential-histogram- and texture-based features are described in the Image Biomarker Standardization Initiative (IBSI) [2] .

Feature type (No. features)	Feature
Differential histogram (20)	Mean
	Variance
	Skewness
	Kurtosis
	Median
	10th percentile
	90th percentile
	Mode
	Interquartile range
	Mean absolute deviation
	Robust mean absolute deviation
	Median absolute deviation
	Coefficient of variation
	Quartile coefficient of dispersion
	Entropy
	Uniformity
	Maximum histogram gradient
	Maximum histogram gradient gray level (GL)
	Minimum histogram gradient
	Minimum histogram gradient GL
GL cooccurrence matrix (GLCM) (22)	Joint maximum
	Joint average
	Joint variance
	Joint entropy
	Difference average
	Difference variance
	Difference entropy
	Sum entropy
	Energy
	Contrast
	Inverse difference (homogeneity 1)
	Inverse difference normalized

	Inverse difference moment (homogeneity 2)
	Inverse difference moment normalized
	Inverse variance
	Correlation
	Autocorrelation
	Cluster tendency
	Cluster shade
	Cluster prominence
	Information correlation 1
	Information correlation 2
GL run length matrix (GLRLM) (16)	Short-run emphasis
	Long-run emphasis
	Low-GL run emphasis
	High-GL run emphasis
	Short-run low-GL emphasis
	Short-run high-GL emphasis
	Long-run low-GL emphasis
	Long-run high-GL emphasis
	GL nonuniformity
	GL nonuniformity normalized
	Run length nonuniformity
	Normalized run length nonuniformity
	Run percentage
	GL variance
	Run length variance
	Run entropy
GL size zone matrix (GLSZM) (16)	Small-zone emphasis
	Large-zone emphasis
	Low-GL zone emphasis
	High-GL zone emphasis
	Small-zone low-GL emphasis
	Small-zone high-GL emphasis
	Large-zone low-GL emphasis
	Large-zone high-GL emphasis
	GL nonuniformity
	GL nonuniformity normalized
	Zone size nonuniformity
	Zone size nonuniformity normalized
	Zone percentage
	GL variance
	Zone size variance
	Zone size entropy
Neighboring GL dependence matrix (NGLDM) (14)	Low-dependence emphasis
	High-dependence emphasis
	Low-GL count emphasis
	High-GL count emphasis
	Low-dependence low-GL emphasis
	Low-dependence high-GL emphasis
	High-dependence low-GL emphasis
	High-dependence high-GL emphasis
	GL nonuniformity
	Dependence-count nonuniformity
	Dependence-count nonuniformity normalized

https://doi.org/10.3390/cancers18040619

	Dependence-count variance<br>Dependence-count entropy<br>Dependence-count energy
Neighborhood gray-tone difference matrix (NGTDM) (5)	Coarseness<br>Contrast<br>Busyness<br>Complexity<br>Strength
DVH (36)	Maximum dose (in gray)<br>Minimum dose (in gray)<br>Mean dose (in gray)<br>$D_{5\%}$<br>$D_{10\%}$<br>$D_{15\%}$<br>$D_{20\%}$<br>$D_{25\%}$<br>$D_{30\%}$<br>$D_{35\%}$<br>$D_{40\%}$<br>$D_{45\%}$<br>$D_{50\%}$<br>$D_{55\%}$<br>$D_{60\%}$<br>$D_{65\%}$<br>$D_{70\%}$<br>$D_{75\%}$<br>$D_{80\%}$<br>$D_{85\%}$<br>$D_{90\%}$<br>$D_{95\%}$<br>V_{5Gy}<br>V_{10Gy}<br>V_{15Gy}<br>V_{20Gy}<br>V_{25Gy}<br>V_{30Gy}<br>V_{35Gy}<br>V_{40Gy}<br>V_{45Gy}<br>V_{50Gy}<br>V_{55Gy}<br>V_{60Gy}<br>V_{65Gy}<br>V_{70Gy}

Table S3. Computation parameters of texture matrices used in this study. We set the parameters in accordance with IBSI [2]. The parameters are presented according to the guidelines, with further details available in IBSI.

**Texture matrix**	**Parameter**	**Configuration (abbreviation as in IBSI)**
GLCM	Matrix aggregation	Merged 3D directions (3D:mrg)
	Cooccurrence matrix symmetry	Symmetry (SYM)

	Distance	Chebyshev distance of 1 (δ-∞:1)
	Distance weighting	No weighting (w:1)
GLRLM	Matrix aggregation	Merged 3D directions (3D:mrg)
	Distance weighting	No weighting (w:1)
GLSZM	Matrix aggregation	Calculated from single 3D matrix (3D)
	Linkage distance	Chebyshev distance of 1 (δ-∞:1)
NGLDM	Matrix aggregation	Calculated from single 3D matrix (3D)
	Dependence coarseness	0 (α:0)
	Distance	Chebyshev distance of 1 (δ-∞:1)
	Distance weighting	No weighting (w:1)
NGTDM	Matrix aggregation	Calculated from single 3D matrix (3D)
	Distance	Chebyshev distance of 1 (δ-∞:1)
	Distance weighting	No weighting (w:1)

Supplementary References

1. Herman, G.T.; Zheng, J.; Bucholtz, C.A. Shape-based interpolation. *IEEE Comput. Graph. Appl.* **1992**, *12*, 69–79.
2. Zwanenburg, A.; Leger, S.; Vallières, M.; Löck, S. Image biomarker standardisation initiative. *arXiv* **2016**, arXiv:1612.07003.
3. Vallières, M.; Freeman, C.R.; Skamene, S.R.; El Naqa, I. A radiomics model from joint FDG-PET and MRI texture features for the prediction of lung metastases in soft-tissue sarcomas of the extremities. *Phys. Med. Biol.* **2015**, *60*, 5471–5496.
4. Vallières, M.; Kay-Rivest, E.; Perrin, L.J.; Liem, X.; Furstoss, C.; Aerts, H.J.W.L.; Khaouam, N.; Nguyen-Tan, P.F.; Wang, C.S.; Sultanem, K.; et al. Radiomics strategies for risk assessment of tumour failure in head-and-neck cancer. *Sci. Rep.* **2017**, *7*, 10117.

Disclaimer/Publisher's Note: The statements, opinions and data contained in all publications are solely those of the individual author(s) and contributor(s) and not of MDPI and/or the editor(s). MDPI and/or the editor(s) disclaim responsibility for any injury to people or property resulting from any ideas, methods, instructions or products referred to in the content.

https://doi.org/10.3390/cancers18040619
